# Evaluation of *in vitro* starch digestibility and chemical composition in pasta former foods

**DOI:** 10.3389/fvets.2022.1049087

**Published:** 2022-11-14

**Authors:** Alessandro Vastolo, Monica I. Cutrignelli, Francesco Serrapica, Dieu donné Kiatti, Antonio Di Francia, Felicia Masucci, Serena Calabro

**Affiliations:** ^1^Department of Veterinary Medicine and Animal Production, University of Naples Federico II, Naples, Italy; ^2^Department of Agricultural Science, University of Naples Federico II, Naples, Italy

**Keywords:** former foods, dry-pasta leftover, alternative feeds, energy content, *in vitro* starch digestibility, barley, pig nutrition

## Abstract

Former food products include various leftovers from the food industry which, although they have lost values for human consumption, could be safely used for livestock, thus limiting environmental impact of food waste, and reducing feeding costs. The aim of this study was to investigate the nutritional characteristics of different types of former foods from pasta industry. Four types of dry pasta refusal (wholemeal, semolina, purple, and tricolor) and whole barley grain (control) were analyzed for chemical composition and *in vitro* starch digestibility; the energy content was also estimated. For each product type, samples collected in three different times at a pasta plant were analyzed. All products showed higher (*p* < 0.001) protein contents and lower (*p* < 0.001) fat contents than barley. The amount of NDF varied between the samples (*p* < 0.001), while all samples reported high starch content (>60% DM). The energy content was higher (*p* < 0.05) in pasta former food compared with whole barley grain. Purple pasta showed different *in vitro* starch digestibility compared to the other former foods (*p* < 0.001). However, all products showed higher values of resistant starch, whereas barley was mainly composed by slowly digestible starch. The results indicated that dry pasta former foods could be suitable energy sources for feeding pig, but their inclusion in diets must consider the slow digestibility.

## Introduction

In recent years, global attention food losses and waste has grown enormously (1). The United Nations' Sustainable Development Goals (SDGs), reflecting the increased awareness of the problem, set a 50% reduction in global per capita food waste at the retail and consumer levels to reduce food losses along and supply chains by 2030 (2). Indeed, approximatively 931 million tons of food waste were generated in 2019 (61% households, 26% food service, and 13% retail). Consequently, food waste is a waste of resources, time and money, and the environmental impact of food production is of no benefit to feeding people (2). There are different terms used to refer the different food surpluses, such as food losses, food waste, and former foods products (FFPs). Former foods products can be used to feed animals, which is not a form of waste treatment; whereas food waste can be further processed to return nutrients to the soil, extract energy and generate heat, but cannot re-enter the food chain (3). Furthermore, according to the European Catalog of Feed Materials, “ex-food” or “FFPs” are defined as foodstuffs, other than catering waste, that have been produced in full compliance with European food legislation, but are no longer intended for human consumption for practical or logistical reasons or due to production or packaging problems, which are unlikely to cause any health risks when FFPs are used as feed (4). In this regard, former foods together with co-products could be a valuable ingredient for animal feed to reduce the environmental impact of waste and promote a more environmentally suitable animal husbandry (5, 6). Former food products have been suggested as one of the categories with great potential as alternative ingredients for animal diet (7). FFPs include various leftovers from the food industry: pasta, bread, cereals, savory snacks, biscuits, cakes, and protein bars. These foods are rich in sugar, starch, protein, oil, or fat, which give them a high energy content (8). Although former foods are already used in animal nutrition their potential as feedstuffs has not been fully exploited, considering that only 3.3% of former foods are used out of the total amount of food waste produced (3). The production of dry pasta is a core activity of the Italian food industry. Currently, around 22% of 14.3 million metric tons of pasta produced worldwide is produced in Italy (9). Italy also has the world's highest consumption of pasta, at around 23.5 kg per capita per year (9). Its low cost, long shelf life, and versatility are behind the popularity of pasta and the main driver of the expansion of consumption (10). In the last decade, pasta production in the European Union has increased from 4,544 to 4, 752 kton/year, with a corresponding growth in pasta consumption from 3,315 to 3,637 kton/year (11). According to Italian law (President Decree No 187/2001), “dry pasta” must be produced by drawing, rolling, and drying a dough prepared with durum wheat semolina (*Triticum durum* Desf.) and water. However, to meet the needs of rapidly expanding market, the pasta sector has evolved over the years by increasing production efficiency, on the one hand, and by improving the hygienic, sensory and nutritional qualities of products on the other (12). Whole grain, multigrain, and vegetable-enriched pasta represent some of the product innovations that have gained a large market share internationally (13–15). As with other agro-industrial supply chains, the expansion of production has exposed the pasta supply chain to some sustainability problems, mainly related to high use resource and high rate of loss and waste produced during production process. In European and North American countries, pasta loss and waste account for about 35% of the total pasta produced, most of which occurs at the processing and retail stages (16, 17). As far as Italy is concerned, most of the waste from pasta supply chain is currently destinated to landfills, incinerators, or composting (18). In this scenario, using dry pasta scraps as alternative ingredients in the diet of farm animals can be an attractive option for simultaneously reducing both food waste and conventional feed requirements. Indeed, dry pasta waste have little variability in terms of chemical composition and a long shelf life due to its low water content.

As far as known, there is a little published data on the use of dry pasta leftover in animal nutrition, which underlines how recycling inedible pasta as feeds has attracted little interest compared to other by-products of food industry (8). In addition to being limited, the available literature (19–24) refers to generic dry semolina pasta, neglecting leftover derived from new or alternative production chains. Therefore, this study was designed to investigate the nutritional characteristics, in terms of chemical composition and *in vitro* starch digestibility, of different types of dry pasta former food products for their potential use in pig feeding plans. Moreover, the nutritional characteristics of the samples were compared with those of whole barley grain, which is one of the most widely used livestock energy feeds for swine.

## Materials and methods

### Sampling procedures and chemical analyses

In a pasta production plant located in Campania, a region in southern Italy, four types of pasta former foods (pFF) were collected, as illustrated in Table 1.

**Table 1:** Pasta former foods (pFF) samples.

**Items**	**Type**	**Composition**
pFF1	Whole meal pasta	Whole durum wheat semolina pasta
pFF2	Semolina pasta	Durum wheat semolina pasta
pFF3	Purple pasta	Whole durum wheat semolina pasta with dehydrated carrot
pFF4	Tricolor pasta	Semolina pasta with dehydrated vegetables (spinach and tomato)

For each type, three samples of ~1 kg each were collected at three different times at least 1 month apart. All pFF samples and barley grain (Hordeum vulgare L. var. *Astartis*, as control) were ground in a laboratory mill (Brabender Wiley mill, Brabender OHG Duisburg, Germany) to 1 mm and analyzed as specified by the Association of Official Analytical Chemists (25) for dry matter (DM; method 930.15), ash (method 942.05), crude protein (CP; method 976.05), ether extract (EE; method 954.02). Analyses of structural carbohydrates [neutral detergent fiber (NDF), acid detergent fiber (ADF) and acid detergent lignin (ADL)] were performed by using an Ankom 220 fiber analyser (ANKOM™ Technology, Fairport, NY, USA). A heat-stable amylase (activity 17.400 Liquefon units/mL, Ankom Technology) was used for NDF procedure. Both NDF and ADF are expressed net of residual ash. The starch content was determined by polarimetry (Polax 2L, Atago, Tokyo, Japan) according to Ewers' method as described by the standard ISO 6493 (26). Non-structural carbohydrates (NSC) were calculated as 100 – (%NDF + %CP + %EE + %Ash) (27). Digestible energy (DE) and metabolizable energy (ME) for pig of the PFFs were calculated using the equations proposed by Noblet and Perez (28) and NRC (29):


$$\begin{array}{l} \text{DE }(\text{kcal/kg})=\text{4}.1\text{68 }(\text{91 x Ash})+(\text{19 x CP})\\ \;+(\text{39 x EE})-(\text{36 x NDF})\\ \text{ME }(\text{kcal/kg})=\text{ DE}-(\text{68 x CP}) \end{array}$$

### *In vitro* starch digestibility

According to Englyst et al. (30), digestibility of starch was analyzed to determine the following fractions: free glucose (FS, 0 min), rapidly digestible starch (RDS, within 20 min of incubation), slowly digestible starch (SDS, between 20 and 120 min) and resistant starch (RS, >240 min) not further hydrolysed. The ground samples were incubated with a solution of pepsin EC (Sigma- Aldrich P-7000) and guar gum in HCl 0.05 mol/L for 30 min at 37°C in a water bath with constant stiring. The enzyme mixture contained 30 g of pancreatin (Sigma-Aldrich P-7545), amyloglucosidase (Megazime E-AMGDF), and invertase (Sigma-Aldrich P-57629). To stop starch digestion, absolute ethanol was added to 1 ml of solution and released glucose was measured calorimetrically at 540 nm as stated on the package insert of the glucose oxidase kit (Sigma-Aldrich GAGO20). The values of total starch (TS), Rapidly Digestible Starch (RDS), Slowly Digestible Starch (SDS), and Resistant Starch (RS) were calculated using the values of glucose released after 20 (G20), 120 min (G120), FG (free glucose) and TG (total glucose) as reported by Englyst et al. (30).


$$\begin{array}{l} \text{ TS}=(\text{TG }-\text{FG})\text{ x }0.9\\ \text{ RDS}=(\text{G20}-\text{FG})\text{ x }0.9\\ \text{SDS}=(\text{G120}-\text{G20})\text{ x }0.9\\ \;RS=(\text{TG}-\text{G120})\text{ x }0.9 \end{array}$$

### Statistical analysis

Chemical composition and *in vitro* digestibility data were analyzed by one-way ANOVA (JMP^®^, Version 14 SW, SAS Institute Inc., Cary, NC, USA, 1989–2019) using substrate as fixed factor. The significance level was checked using Tukey's HSD test at *p* < 0.01 and *p* < 0.05. *Post hoc* Dunnett test was performed to observe the differences between barley grain (control) and pFFs samples. The statistical comparison Shapiro-Wilk test for normally distributed data was performed.

## Results

**Figure 1:**
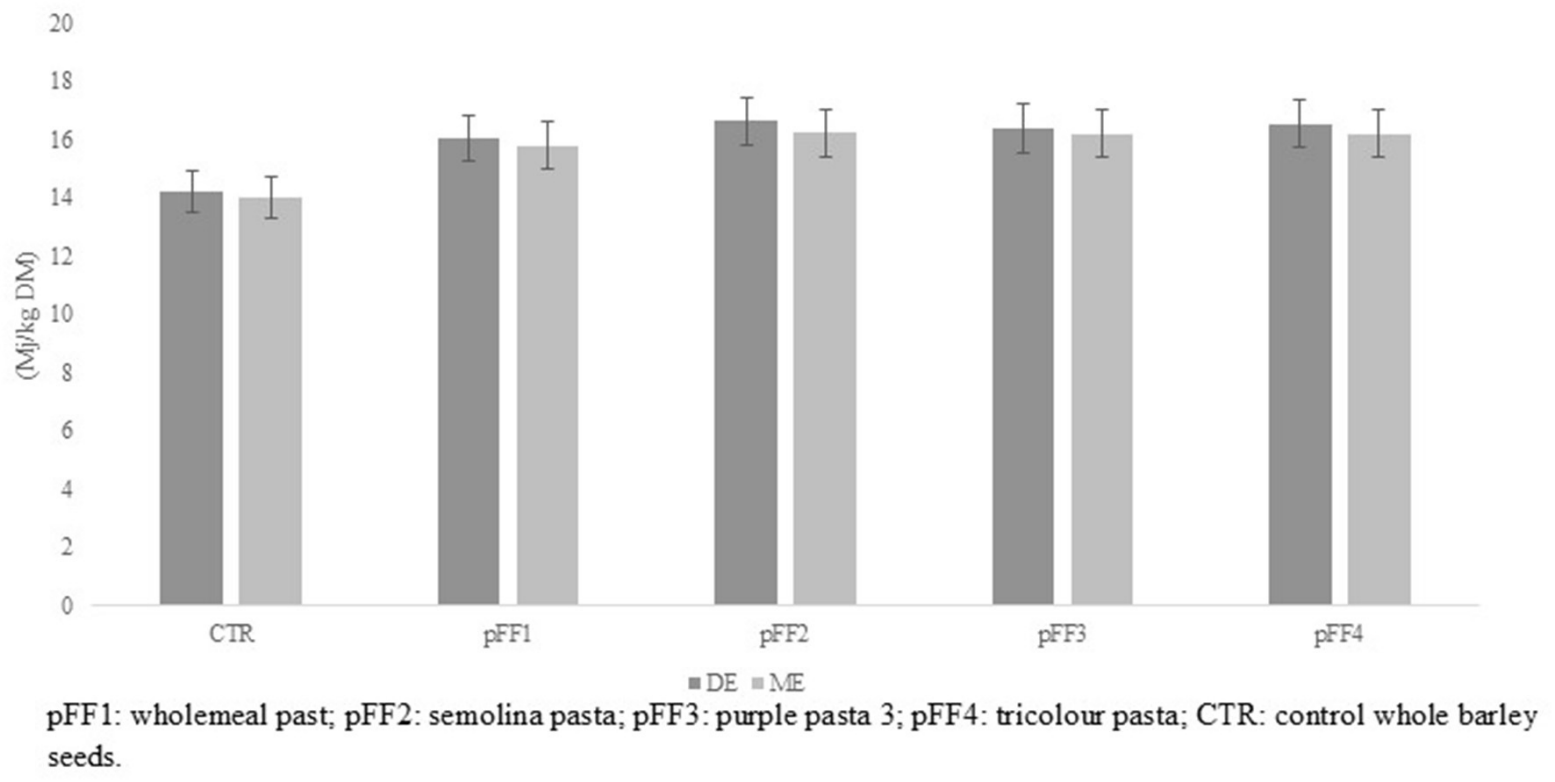
Digestible (DE) and metabolizable (ME) energy content of barley (control) and pasta former foods (pFFs).

The chemical composition of the pFFs and the reference barley are shown in Table 2. The pFFs differed significantly (*p* < 0.001) in chemical characteristics. In particular, pFF1 obtained from whole wheat flour showed the lowest levels (*p* < 0.001) of starch and NSC and, in contrast, the highest contents (*p* < 0.001) of structural carbohydrates (NDF, ADF, and ADL) and EE. Opposite characteristics were shown by the semolina pasta pFF2, which also presented a lower CP level than pFF3 and pFF4. The latter products showed intermediate characteristics between pFF1 and pFF2. The pFF1 pasta also showed the lowest value (*p* < 0.01) of metabolizable energy due to the higher amount of structural carbohydrates and the lower percentages of starch and NSC. Dunnett's test showed that pasta former foods were richer in CP and starch and poorer in lignin and ether extract than barley. As an overall result, barley showed a lower (*p* < 0.001) level of ME (*p* < 0.001) compared to pFFs (Figure 1).

**Table 2:** Chemical composition of the different pasta former food and whole barley seeds (% DM) (*n* = 4).

	**pFF1**	**pFF2**	**pFF3**	**pFF4**		**CTR vs. pFF samples**				**MSE**
					**CTR**	**pFF1**	**pFF2**	**pFF3**	**pFF4**	
DM	88.9	89.4	88.1	89.5	89.6	NS	NS	*	NS	0.15
CP	15.7^A^	12.6^B^	14.5^A^	15.1^AB^	10.5	***	**	***	***	0.09
NDF	12.5^A^	2.08^C^	1.94^C^	11.4^B^	11.9	NS	***	***	NS	0.07
ADF	2.21^A^	0.56^B^	0.38^B^	1.31^AB^	7.87	***	***	***	***	0.06
ADL	0.32^A^	0.12^C^	0.22^B^	0.18^B^	2.04	***	***	***	***	0.002
Starch	68.0^C^	72.9^A^	71.1^B^	70.4^B^	65.1	***	***	***	***	0.08
NSC	69.3^D^	84.2^A^	82.3^B^	72.1^C^	71.8	**	***	***	NS	0.19
EE	0.66^A^	0.20^BC^	0.16^C^	0.23^B^	2.28	***	***	***	***	1E-4
Ash	1.84^A^	0.86^D^	1.04^C^	1.19^B^	3.34	***	***	***	***	4E-4

pFF1, whole meal past; pFF2, semolina pasta; pFF3, purple pasta; pFF4, tricolor pasta; CTR, control whole barley seeds; DM, dry matter; CP, crude protein; NDF, neutral detergent fiber; ADF, acid detergent fiber; ADL, acid detergent lignin; NSC, not structural carbohydrates; EE, ether extract; A, B, C, D: p < 0.01 in comparison between tested pasta former foods. ^*^, ^**^, ^***^, NS: p < 0.05, p < 0.01, p < 0.001, not significant, respectively; MSE, mean square error.

**Figure 2:**
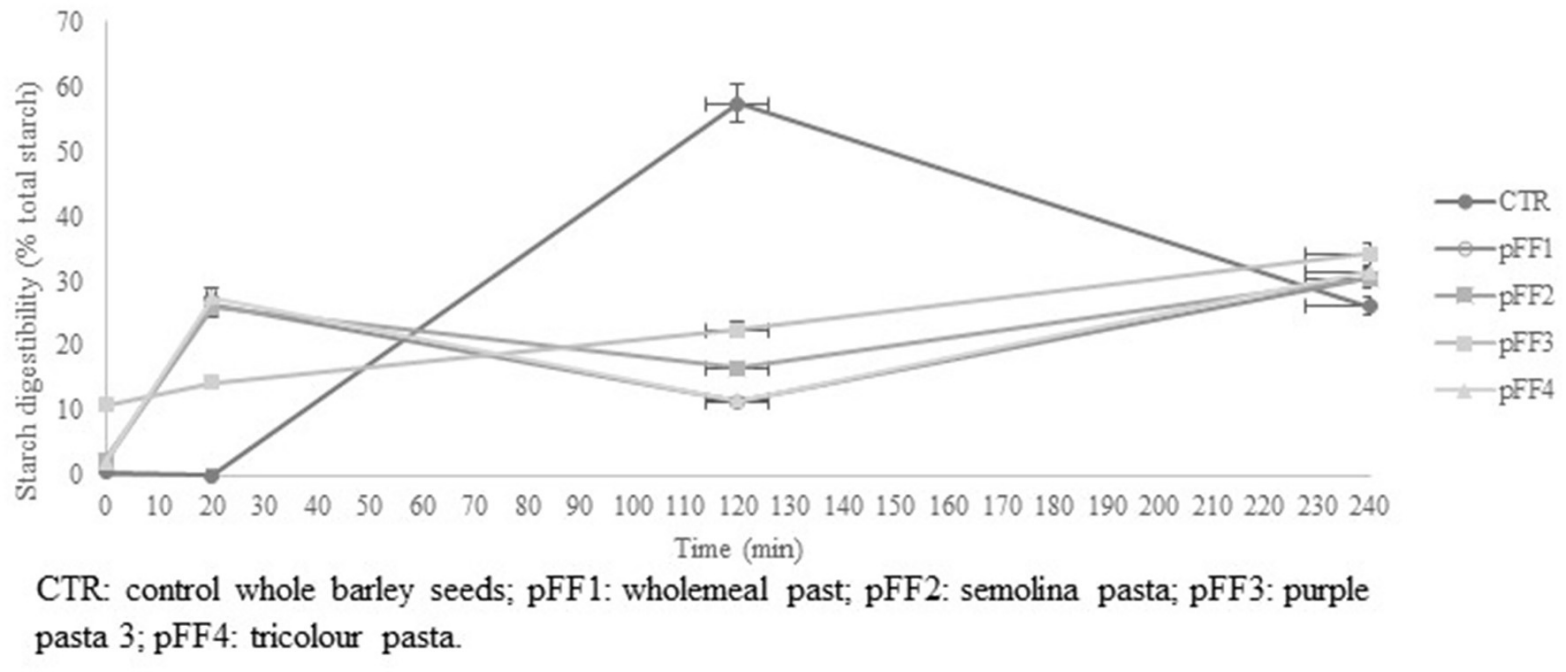
*In vitro* starch digestibility over time of pFF and barley seeds (control).

The *in vitro* starch digestibility is shown in Table 3. Purple pasta (pFF3) presented the highest level (*p* < 0.001) of free glucose (FG), SDS and RS, but the lowest percentage (*p* < 0.001) of RDS. Comparison with reference barley showed that all pFF samples had higher level (*p* < 0.001) of FG, RDS, and RS, while barley had the highest amount (*p* < 0.001) of SDS. Concerning starch digestibility over time (Figure 2), purple pasta (pFF3) showed linear starch digestibility during incubation and presented the highest level of free glucose (FG) and the lowest percentage of rapidly digestible starch (RDS). In contrast, pFF2, and pFF4 showed unpredictable trends, as the percentage of starch degradation increased in the first 20 min and then reached the minimum level at 120 min. Therafter, the percentage of degraded starch increased again. The reference barley showed a low digestibility rate in the first few minutes and then increased and reached the maximum rate at 120 min.

**Table 3:** *In vitro* starch digestibility of pasta former foods (% of total starch) (*n* = 4).

	**pFF1**	**pFF2**	**pFF3**	**pFF4**		**CTR vs. pFF samples**				**MSE**
					**CTR**	**pFF1**	**pFF2**	**pFF3**	**pFF4**	
FSG	3.43^B^	3.38^B^	15.4^A^	2.97^B^	0.87	^***^	^***^	^***^	^***^	0.05
RDS	38.6^A^	35.6^A^	20.2^B^	39.1^A^	0.11	^***^	^***^	^***^	^***^	5.43
SDS	11.7^B^	17.8^B^	31.7^A^	16.4^B^	98.2	^***^	^***^	^***^	^***^	2.99
RS	46.7^ab^	44.1^b^	48.0^a^	44.8^b^	1.71	^***^	^***^	^***^	^***^	0.48

pFF1, whole meal past; pFF2, semolina pasta; pFF3, purple pasta; pFF4, tricolor pasta; CTR, control whole barley seeds; FSG, free sugar glucose; RDS, rapidly digestible starch; SDS, slowly digestible starch; RS, resistant starch. A, B, and a, b: p < 0.01 and 0.05, respectively in comparison between tested pasta former foods.

*** p < 0.001; NS, not significant; MSE, mean square error.

## Discussion

The re-use of the food industry leftovers in animal feeding is considered as a virtuous pathway to promote food safety by minimizing losses along the supply chains (3). However, the use of ex-foods in the standard diet formulation is still limited due to the wide range of available co-products and the lack of knowledge of their specific nutritional characteristics (8). Updating the differences between the type of leftovers from different supply chains is the first step to fill existing gaps and to promote a rational recycling of ex-food as low cost-ingredients by the feed industry (31). This need is greatest when leftovers are recovered from multi-ingredients food production chains, such as the modern pasta industry. In this study four different types of former foods from pasta were evaluated and compared to whole barley seeds as reference feed. Semolina and whole-wheat pasta are a typical product, while tricolor and purple products represent a new trend in the pasta industry. Since barley is widely used in pig feeding plan, the results will be discussed mainly in relation to this species.

With regard to chemical composition and energy content of both pFFs and barley grain appeared to be quite in line with data reported in the literature reports (7, 32, 33). Higher protein levels were observed for pFFs compared to barley, their amounts do not appear to be sufficient to meet the specific requirements of pigs, both in terms of quantity and quality, according to their wheat-based formulation (34, 35). The higher NDF content of pFF1 and pFF4 compared to the other products is comparable to that of the control and is clearly attributable to the ingredients used in the production of these types of pasta. In fact, unlike semolina and purple pasta, pFF1 and pFF4 are produced with whole wheat and, consequently, have a higher content of structural carbohydrates. This is a positive aspect as the presence of fiber in swine rations can reduce the excretion of pollutants and improve the absorption of micronutrients (36). The pFF samples were particularly low in lipids, and their energy content is essentially due to the starch content, which is the main energy source in the monogastric diet (37). As a general consideration, the non-structural carbohydrate, starch, and energy content of pFFs are comparable to the main carbohydrate sources used in swine diets (38). All samples tested showed low levels of FG, except for pFF3 that, in addition, showed a different starch digestibility, had a lower RDS, and a higher SDS compared to the products obtained from whole meal wheat flour (pFF1), and purple pasta (pFF3). It appears that the presence of dehydrated carrots in purple pasta has influenced the digestibility of starch *in vitro* (39). Bufler (40) reported that carrots contain high amounts of starch, which when mobilized can lead to a significant increase in sugar content. All pFF samples showed a high level of resistant starch (RS) probably related to the size and shape of the granules (41). According to their X-ray diffraction pattern, the starch granules have been characterized as type A, B, or C. Processed foods (e.g., biscuits, pasta) in which the starch is incompletely gelatinized (42, 43), mainly present B or C starch granules that are more resistant to digestion by pancreatic amylase. This resistance to hydrolysis affects the digestibility of starchy foods that are eaten raw. However, as reported by Sandhu and Lim (44), colon health could be improved by the higher percentage of RS due to its fermentability in the large intestine. Control barley showed a completely different starch digestibility *in vitro*, with a high percentage of slowly degradable starch. Barley, particularly var. *Astartis*, is rich in β-Glucans, which form a large part of the cell wall of the endosperm of cereal seeds and are largely indigestible (31). Consequently, this cereal is rich in soluble dietary fiber, which may ensure a slower intestinal transit (36). Furthermore, many sources of dietary fiber influence digestion and fermentation, reducing pathogens and improving intestinal barrier function (45). In this sense, RS-rich feeds resist digestion in the stomach and enzymatic hydrolysis in the small intestine, being available for fermentation in the large intestine, resulting in the production of short-chain fatty acids (SCFA) (46). A high production of SCFA, particularly butyrate, may have an impact on pig health providing energy to enterocytes and maintaining gut barrier integrity (47). Furthermore, feedstuffs rich in resistant starch have the potential to improve health status by influencing postprandial glycemia and insulinemia and increasing minerals absorption (48). Ottoboni et al. (49) measured the hydrolysis index (HI), the predicted glycemic index (pGI), and the time course in carbohydrate digestion (*k*) in former foods compared to common cereals. The authors indicated that all parameters related to carbohydrate digestion were consistently higher in ex-food compared to conventional cereal-based ingredients. Specifically for dry pasta, this may also be due to the low-temperature extrusion process (around 50°C) to which pasta is subjected which may affect its digestibility (12). Furthermore, according to Tretola et al. (50) former food products such as bakery products and confectionary products can replace up to 30% of conventional cereal grains commonly used in pig diets without negatively affecting growth performance and nutritional status of piglets after weaning. Furthermore, the partial substitution of unprocessed starch with processed starch can increase the feed digestibility due to the nature of former foods, originally intended for human consumption and subjected to a wide range of processing techniques.

## Conclusion

In recent years, it has become necessary to find alternative sources of animal nutrition due to increasing global demand for food and feed compared to traditional feeds. In this scenario, dry pasta residues could represent an alternative, given the little variable chemical composition. In fact, the latter was quite similar between the pasta-former foods, while the *in vitro* digestibility of starch differed, probably as the pasta processing modified starch structure affecting the enzymatic utilization. Overall, the examined pasta's former foods seem suitable in rations for swine as an energy source with high starch content as an alternative to traditional cereals, but the different starch digestibility must be considered. Further *in vivo* studies are needed to evaluate the right amount of these ingredients in a balanced diet.

## Data availability statement

The raw data supporting the conclusions of this article will be made available by the authors, without undue reservation.

## Author contributions

MC and SC conceptualized and supervised the study. AV, FS, and DK conducted the formal analysis. AV, FM, AD, and FS contributed to the methodology and data curation and wrote the original draft. AV and DK conducted the statistical analysis. FM, MC, SC, AV, and FS reviewed and edited the manuscript. All authors contributed to the article and approved the submitted version.

## Conflict of interest

The authors declare that the research was conducted in the absence of any commercial or financial relationships that could be construed as a potential conflict of interest.

## Publisher's note

All claims expressed in this article are solely those of the authors and do not necessarily represent those of their affiliated organizations, or those of the publisher, the editors and the reviewers. Any product that may be evaluated in this article, or claim that may be made by its manufacturer, is not guaranteed or endorsed by the publisher.
